# Anticataractogenesis and Antiretinopathy Effects of the Novel Protective Agent Containing the Combined Extract of Mango and Vietnamese Coriander in STZ-Diabetic Rats

**DOI:** 10.1155/2017/5290161

**Published:** 2017-08-20

**Authors:** Jintanaporn Wattanathorn, Paphaphat Thiraphatthanavong, Wipawee Thukham-mee, Supaporn Muchimapura, Panakaporn Wannanond, Terdthai Tong-un

**Affiliations:** ^1^Integrative Complementary Alternative Medicine Research and Development Center, Khon Kaen University, Khon Kaen 40002, Thailand; ^2^Department of Physiology, Faculty of Medicine, Khon Kaen University, Khon Kaen 40002, Thailand

## Abstract

The novel protectant against diabetic cataract and diabetic retinopathy is currently required due to the increased prevalence and therapeutic limitation. Based on the advantage of polyphenol on diabetic eye complications, we hypothesized that the combined extract of mango seed Vietnamese coriander (MPO), a polyphenol-rich substance, should possess anticataractogenesis and antiretinopathy in streptozotocin- (STZ-) diabetic rats. MPO at doses of 2, 10, and 50 mg/kg·BW were orally given to STZ-diabetic rats for 10 weeks. Lens opacity was evaluated every week throughout a study period whereas the evaluation of cataract severity and histological changes of both rat lens epithelium and retina together with the biochemical assays of oxidative stress status, aldose reductase, p38MAPK, ERK1/2, and VEGF were performed at the end of experiment. Our data showed that MPO improved cataract and retinopathy in STZ-diabetic rats. The improved oxidative stress status and the decreased p38MAPK, ERK1/2, and VEGF were also observed. Therefore, anticataractogenesis and antiretinopathy of MPO might occur partly via the decreased oxidative stress status and the suppression of aldose reductase, p38MAPK, ERK1/2, and VEGF. This study points out that MPO is the potential candidate protectant against diabetic cataract and diabetic retinopathy. However, the exploration for possible active ingredient (S) still requires further researches.

## 1. Introduction

At present, the prevalence of diabetic eye complications such as diabetic cataract and diabetic retinopathy, the leading causes of an acquired blindness, is increasing due to the survival of diabetic patients. In recent years, a pile of evidence has clearly revealed that oxidative stress contributes an important role in the pathophysiology of diabetic cataract and diabetic retinopathy [1–3]. It has been reported that both lens and retina are very much susceptible to oxidative stress because of the limited capacity of antioxidant system in the lens together with the polyunsaturated fatty acid-rich and the highest oxygen uptake and glucose oxidation of retina [4]. Oxidative stress can induce cataract and retinopathy by numerous pathways including the activation of polyol pathway, vascular endothelial growth factor (VEGF), and mitogen-activated protein kinase (MAPK) [5–7]. Based on the crucial roles of oxidative stress mentioned earlier, the possibility to prevent or to improve diabetic cataract and diabetic retinopathy by antioxidant has been raised.

Recently, numerous studies have demonstrated that the phenolic compounds show a wide range of therapeutic potentials such as antioxidant, anti-inflammation, and antidiabetic effects [8]. In addition, it has been demonstrated that polyphenolics such as quercetin and the substances which are rich in polyphenolic compounds can improve diabetic cataract and diabetic retinopathy [8–11]. Our pilot study has demonstrated that the combined extract of mango seed and Vietnamese coriander contains high contents of phenolic compounds and exhibits both an antioxidant and an aldose reductase suppression effects. In addition, the data concerning acute toxicity of the combined extract of mango seed and Vietnamese coriander show that the lethal dose 50 (LD50) is more than 5 g/kg BW which indicates that it is practically safe. Therefore, the potential benefits of the combined extract of mango seed and Vietnamese coriander on diabetic cataract and diabetic retinopathy have been considered. Since no scientific data concerning this issue was available, this study aimed to determine the preventive effect of the combined extract of mango seed and Vietnamese coriander on cataract and retinopathy in streptozotocin- (STZ-) diabetic rat model.

## 2. Materials and Methods

### 2.1. Plant Material and Extract Preparation

The seed of ripe mango or *Mangifera indica* L. (Nam Dok Mai) and aerial parts of Vietnamese coriander or *Polygonum odoratum* L. were used as the natural resources in this study. They were harvested in May 2015 and prepared as 50% hydroalcoholic extracts by maceration technique at ratios of 2 : 5 and 2 : 15 (weight : volume), respectively. The samples were macerated at room temperature for 3 days. The yielded extracts were concentrated by lyophilization and kept at 4°C for further study. The percentage yields of the seed of *Mangifera indica* L. and aerial parts of *Polygonum odoratum* L. extracts were 43.48 and 8.89, respectively. The combined extract of *Mangifera indica* and *Polygonum odoratum* (MPO) was prepared at a ratio of 1 : 5. (This ratio was selected based on its advantage in vitro potential on antioxidant and aldose reductase inhibition.) The finger print chromatogram of the combination extract or MPO was carried out via gradient high-performance liquid chromatography (HPLC) system consisting of 515 HPLC pump and 2998 photodiode array detector of Waters Company, USA. Chromatographic separation was performed using Purospher® STAR, C-18 encapped (5 *μ*m), LiChroCART®250-4.6, and HPLC-Cartridge, Sorbet Lot No. HX255346 (Merck, Germany). Two mobile phases consisting of 2.5% acetic acid in deionized (DI) water and methanol were used to induce gradient elution. The injection volume was 20 *μ*l and the flow rate was 1.0 ml/min. During a 30-minute analysis via HPLC, the solvent gradient was programmed as follows: 0 min, 10% A; 17 min, 70% A; 18 min, 100% A; 22 min, 100% A; 25 min, 50% A; 27 min, 10% A; and 30 min, 10% A. Data analysis was performed using EmpowerTM 3. Gallic acid monohydrate ≥ 99% (HPLC), Sigma-Aldrich, and quercetin ≥ 95% (HPLC), Sigma-Aldrich, were used for standard.

### 2.2. Experimental Design

The experimental animals in this study were male Wistar rats from the National Laboratory Animal Center, Salaya, Nakhon Pathom, at the weight of 180–220 g (*n* = 8 per group). All experimental protocols used in this study had been approved by the Institutional Animal Care and Use Committee Khon Kaen University, Thailand (AEKKU-NELAC 8/2558). All rats were divided into various groups as follows:

Group I: control group—all rats in this group were administered citrate buffer, a vehicle of STZ.
Group II: DM +  vehicle group—all experimental animals were induced diabetes mellitus via single injection of STZ and received distilled water or vehicle of the extract.
Groups III-V: DM +  the combination extract of *Mangifera indica* L. and *Polygonum odoratum* L. (MPO)—all rats were induced diabetes mellitus and received MPO at doses of 2, 10, and 50 mg/kg BW, respectively.

In groups II–V, all rats were induced diabetes cataract and retinopathy by a single injection of STZ which was dissolved in citrate buffer (pH 4.5) at a dose of 55 mg/kg BW. The rats which showed the blood sugar levels more than 250 mg/dl were recruited for further study. All rats were treated with the assigned interventions once daily since 3 days after the injection of STZ and maintained for 10 weeks. The evaluation of lens opacity was performed every week throughout the study period using slit lamp microscope. At the end of study, lens were collected and determined histomorphology, vascular endothelial growth factor (VEGF), mitogen-activated protein kinase (MAPK), aldose reductase (AR), and oxidative stress including malondialdehyde (MDA) level and the activities of superoxide dismutase (SOD), catalase (CAT), and glutathione peroxidase (GPx) enzymes in lens.

### 2.3. Determination of Fasting Blood Glucose Level

After fasting overnight, blood was collected from rat tails. Fasting blood glucose measurement was performed by using ACCU-CHEK. This process was performed at the first week and every 5 weeks throughout the study period.

### 2.4. Cataract Evaluation

Cataract evaluation was performed via slit lamp microscope (Dioptrix-Hawk Eye; France) by a trained observer who was blind to the treatment every week. The cataract severity was graded according to the method of Suryanarayana and coworkers [12] as follows:

Stage 0: clear lenses with no vacuole are observed.
Stage 1: vacuoles cover approximately one-half of the surfaces of the anterior pole forming a subcapsular cataract.
Stage 2: some vacuoles have disappeared and the cortex exhibits a hazy opacity.
Stage 3: a hazy cortex remains and dense nuclear opacity is present.
Stage 4: a mature cataract is observed as a dense opacity in both cortex and nucleus.

The opacity index was presented. It was calculated from the following formula:

(1) $$\begin{array}{c} Opacity\;index=\frac{number\;of\;eyes\;in\;each\;stage\times stage\;of\;the\;eye}{total\;number\;of\;eyes\;within\;group}. \end{array}$$

### 2.5. Homogenate Preparation

At the end of experimental period, lens were collected and homogenized in 10 volume of 0.1 M phosphate buffer, pH 7.4, containing 1 mM EDTA. Then, the homogenate was subjected to a 10,000*g* centrifugation for 1 hour at 4°C. The supernatant was separated and used for the determination of biochemical parameters.

### 2.6. Determination of Malondialdehyde (MDA) Level

Thiobarbituric acid reacting substances (TBARS) were used for measuring the malondialdehye (MDA) level, a lipid peroxidation in rat lens. In brief, 100 *μ*l of sample was mixed with the solution containing 100 *μ*l of 8.1% (*w*/*v*) sodium dodecyl sulphate, 750 *μ*l 20% (*v*/*v*) acetic acid (pH 3.5), and 750 *μ*l of 0.8% thiobarbituric acid (TBA). The solution was heated in a water bath at 95°C for one hour and cooled immediately under running tap water. Then, 500 *μ*l chilled water and 2500 *μ*l of butanol and pyridine [15 : 1 *v*/*v*] were added to each tube and mixed thoroughly with vortex. Then, the solution was subjected to an 800*g* centrifugation for 20 min. The upper layer was separated and measured the absorbance at 532 nm. 1,3,3-Tetra ethoxy propane (TEP) was used as the reference [13]. The level of MDA was expressed as U/mg protein.

### 2.7. Superoxide Dismutase (SOD) Assay

Superoxide dismutase activity was performed based on nitrobluetetrazolium (NBT) reduction assay. In this assay, the xanthine-xanthine oxidase system was used as a superoxide generator. In brief, 20 *μ*l of sample was mixed with the reaction mixture containing 57 mM phosphate buffer solution (KH_2_PO_4_), 0.1 mM EDTA, 10 mM cytochrome C solution, 50 *μ*M of xanthine solution, and 20 *μ*l of xanthine oxidase solution (0.90 mU/ml) at 25°C. The optical density was measured at 415 nm. A system devoid of enzyme served as the control, and three parallel experiments were conducted [14]. SOD activity was expressed as U/mg protein.

### 2.8. Catalase (CAT) Assay

Catalase activity was assessed based on the ability of the enzyme to break down H_2_O_2_. In brief, 10 *μ*l of sample was mixed with the reaction mixture containing 50 *μ*l of 30 mM hydrogen peroxide (in 50 mM phosphate buffer, pH 7.0), 25 *μ*l of H_2_SO_4_, and 150 *μ*l of KMnO_4_. After mixing thoroughly, the optical density was measured at 490 nm. A system devoid of the substrate (hydrogen peroxide) served as the control. The difference in absorbance per unit time was expressed as the activity. An amount of enzyme required to decompose 1.0 M of hydrogen peroxide per minute at pH 7.0 and 25° is regarded as one unit [15]. The value of CAT activity was expressed as U/mg protein.

### 2.9. Glutathione Peroxidase (GPx) Assay

The glutathione recycling method was used to assay this enzyme by using 5,5′-dithiobis-2-nitrobenzoic acid (DTNB) and glutathione reductase. The reaction between DTNB and GSH gave rise to the generation of 2-nitro-5-thiobenzoic acid and GSSG. GSH concentration was determined by measuring absorbance of 2-nitro-5-thiobenzoic acid, a yellow-colored product, at 412 nm. In brief, a mixture containing a 20 *μ*l of sample and the reaction mixture consisting of 10 *μ*l of dithiothreitol (DTT) in 6.67 mM potassium phosphate buffer (pH 7), 100 *μ*l of sodium azide in 6.67 mM potassium phosphate buffer (pH 7), 10 *μ*l of glutathione solution, and 100 *μ*l of hydrogen peroxide were mixed thoroughly and incubated at room temperature for 10 minutes. Then, 10 *μ*l of DTNB (5,5-dithiobis-2-nitrobenzoic acid) was added, and the optical density at 412 nm was recorded at 25°C over a period of 5 min. GPx activities were expressed as U/mg protein. Each unit is referred to as *μ*mol/min/mg protein [16].

### 2.10. Aldose Reductase (AR) Activity Assay

The spectrophotometric method was used to determine aldose reductase activity. A mixture containing 0.7 ml of phosphate buffer (0.067 mol), 0.1 ml of NADPH (25 × 10^−5^ mol), 0.1 ml of DL-glyceraldehyde (substrate, 5 × 10^−4^ mol), and 0.1 ml of lens supernatant were prepared. Absorbance was recorded against a reference cuvette containing all other components except the substrate, DL-glyceraldehyde. The final pH of the reaction mixture was adjusted to pH = 6.2. The determination was performed after adding the substrate or DL-glyceraldehyde by measuring the decrease in NADPH absorbance at 390 nm over a 4-minute period [17]. The enzyme activity was expressed as (nmol/min/mg).

### 2.11. ERK1/2 Assay

ERK1/2 (ab176641, Abcam®) was evaluated using the colorimetric method of Abcam. 50 *μ*l of all standards and samples was added to a 96-well plate. 50 *μ*l of antibody cocktail that was prepared by combining 3 ml of 10x ERK1/2 (total) capture antibody and 3 ml of 10x ERK1/2 (total) detector antibody was added to each well, and the plate was sealed and incubated for 1 hour at room temperature on a plate shaker set to 400 rpm. Each well was washed 3 times with 350 *μ*l 1x wash buffer. After the last wash, the plate was inverted and blotted against clean paper towels to remove excess liquid. Then, 100 *μ*l of TMB substrate was added and subjected to a 400 rpm shaker in a dark condition for 15 minutes. Finally, 100 *μ*l of stop solution was added to each well, shook for 1 minute to mix it thoroughly, and recorded the optical density at 450 nm.

### 2.12. p38-MAPK Alpha Assessment

p38-MAPK alpha (ab176650, Abcam) was evaluated using the colorimetric method of Abcam. 50 *μ*l of all standards and samples was added to a 96-well plate. 50 *μ*l of antibody cocktail containing 3 ml of 10x p38-MAPK alpha (total) capture antibody and 3 ml of 10x p38-MAPK alpha (total) detector antibody were added to each well. After being sealed, the plate was incubated for 1 hour at room temperature by using an ab400 rpm plate shaker. Each well was washed 3 times with 350 *μ*l 1x wash buffer, inverted, and blotted against clean paper towels to remove excess liquid. Then, 100 *μ*l of TMB substrate was added and subjected to a 400 rpm shaker in a dark condition for 15 minutes. 100 *μ*l of stop solution was added to each well, shook for 1 minute, and recorded the absorbance at 450 nm.

### 2.13. Determination of VEGF

A colorimetric method of Abcam was used for the VEGF (ab100787, Abcam) assessment. A 96-well plate containing 100 *μ*l of all standards and samples was covered and incubated for 2.5 hours at 4°C with gentle shaking. Each well was washed 4 times with 300 *μ*l 1x wash buffer. To remove excess liquid, the plate was inverted and blotted against clean paper towels after the last wash. The administration of 100 *μ*l of 1x biotinylated VEGF detection antibody to each well was performed and incubated for 1 hour at room temperature with gentle shaking. The solution was discarded and repeated the wash. Then, 100 *μ*l of 1x HRP-streptavidin solution was added and incubated for 45 minutes at room temperature with gentle shaking. Then, 100 *μ*l of TMB one-step substrate reagent was added to each well after the repeated wash and incubated for 30 minutes at room temperature in the dark with gentle shaking. Finally, 50 *μ*l of stop solution was added and the absorbance at 450 nm was immediately read.

### 2.14. Histopathological Analysis

The eye balls were removed and fixed in 10% formalin overnight, embedded in paraffin, sectioned at 5 *μ*m thick, and stained with hematoxylin and eosin. The histomorphological changes of the lens were determined by using light microscope. The severity of histomorphological change of lens was graded as a 5-grade score according to the method of Agarwal et al. [18] as described in the following:

Grade 0: presence of anterior epithelium with lens fibers
Grade 1: presence of anterior epithelium, lens fibers and vacuoles
Grade 2: presence of anterior epithelium, lens fibers, vacuoles, and homogenized area
Grade 3: absence of anterior epithelium, presence of lens fibers, vacuoles and homogenized area
Grade 4: presence of lens fibers and homogenized area only.

The retinal histomorphological changes including the total retinal thickness (from the inner limiting membrane to Bruch's membrane), the thickness of the retinal outer nuclear layer, and the number of cells in the ganglion cell layer were also performed. The average of retinal thickness was evaluated using 3 adjacent fields and total five images in each group [19]. The results were showed as mean ± SEM.

### 2.15. Statistical Analysis

All data were presented as mean ± standard error mean (mean ± SEM). The analysis of data was performed using one-way analysis of variance (ANOVA) followed by the post hoc test of LSD via SPSS version 15. Statistical differences were considered at *p* value < .05.

## 3. Results

### 3.1. Phytochemical Screening of MPO Extract

**Figure 1:**
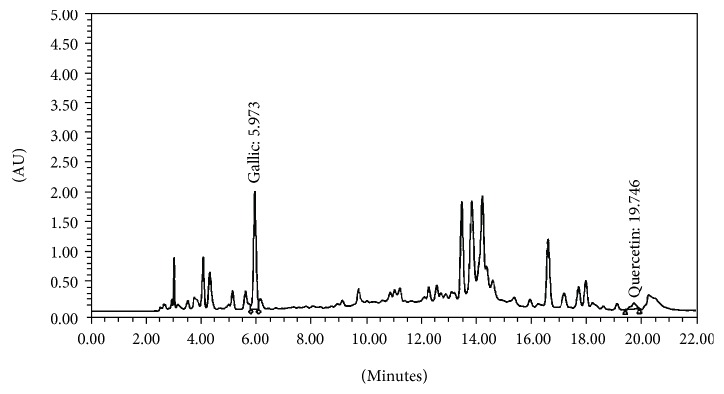
Fingerprint chromatogram of the combination extract of mango seed and aerial parts of Vietnamese coriander (MPO).

The finger print chromatogram of the combination extract or MPO was shown in Figure 1. It was found that the content of total phenolic compounds in the combination extract was 81.96 ± 2.42 mg/l GAE/mg extract. Quercetin and gallic acid were presented at the concentrations of 2.286 mg quercetin equivalent (QE)/100 mg extract and 0.636 mg gallic acid/100 mg extract, respectively.

### 3.2. Blood Glucose Changes

**Figure 2:**
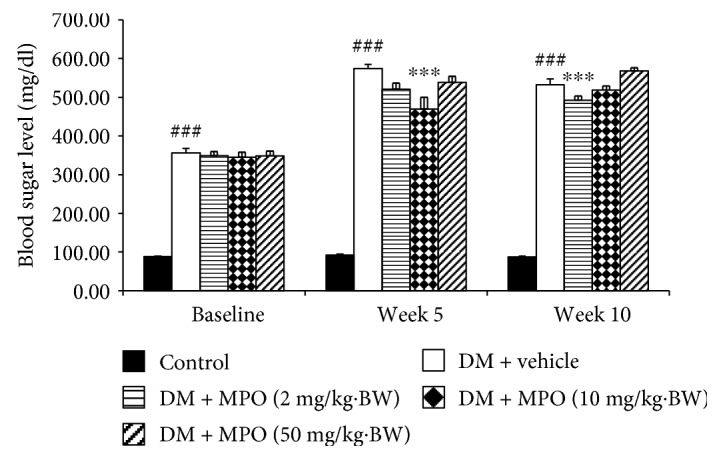
The average fasting blood sugar levels of all groups at various time points (1-week, 5-week, and 10-week intervention period (*N* = 10/group)). ^###^*p* value < .001 compared with the control group. ^∗∗∗^*p* value < .001 compared with diabetic rats which received vehicle (DM + vehicle).

Figure 2 showed that diabetic rats which received vehicle significantly increased blood sugar level throughout a 10 week-study period compared to the control group (*p* value < .001). Baseline data showed no significant differences in the blood sugar level among diabetic groups.

After MPO treatment for 5 weeks, diabetic rats which received MPO at a dose of 10 mg/kg BW significantly decreased blood sugar level compared to diabetic rats with vehicle treatment (*p* value < .001). When the treatment was prolonged to 10 weeks, only diabetic rats which received MPO at a dose of 2 mg/kg BW showed the decreased blood sugar level compared to diabetic rats with vehicle treatment (*p* value < .001). No other significant changes of this parameter were observed.

### 3.3. Anticataract Effect

**Figure 3:**
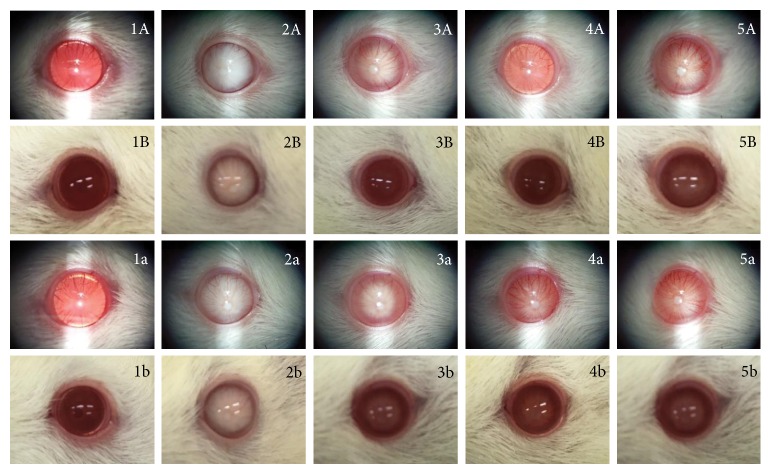
Representative photographs of rat lenses at the end of a 10-week study period. Photographs via slit lamp (A, a). Photographs via camera (B, b). 1: control rat; 2: diabetic rat which received vehicle; 3: diabetic rat which received MPO at a dose of 2 mg/kg·BW; 4: diabetic rat which received MPO at a dose of 10 mg/kg·BW; 5: diabetic rat which received MPO at a dose of 50 mg/kg·BW.

The anticataract effect of MPO was shown in Figure 3 and Table 1. It was found that lens opacity of both eyes of diabetic rats increased. The significant increase of lens opacity was observed since the second week of study period, and this change was presented throughout the study period compared to that in the control group (*p* value < .05, .05, .001, .001, .001, .001, .001, .001, and .001, resp.). At 4 weeks of treatment, diabetic rats which received MPO at doses of 2 and 50 mg/kg BW decreased lens opacity compared to diabetic rats with vehicle treatment (*p* value < .05 all). When the treatment was prolonged further to 5 weeks, lens opacity of diabetic rats which received MPO at all dosage ranges used in this study were markedly decreased when compared to diabetic rats with vehicle treatment (*p* value < .001 all). The significant changes were also observed between the sixth week and the tenth week of the treatment compared to those in the diabetic rats with vehicle treatment (*p* value < .001 all; *p* value < .001 all; *p* value < .001 all; *p* value < .01, .001, and .01,; *p* value < .05, .001, and .05, resp.).

**Figure 4:**
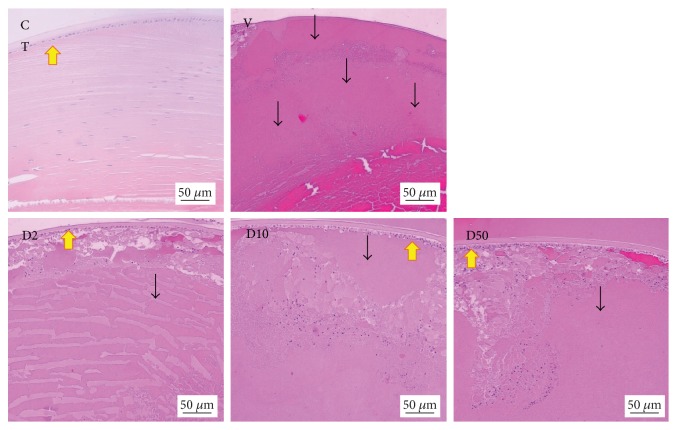
Representative histological photographs of transverse sections of eye balls (5 *μ*m thickness) showing severity of cataract at 10-week study period evaluated under light microscope. The lens tissue sections were stained with H&E. Yellow arrow: anterior epithelium with lens fibers. Black arrow: homogenized area. C: control; V: DM + vehicle; D2: DM + MPO (2 mg/kg·BW); D10: DM + MPO (10 mg/kg·BW); D50: DM + MPO (50 mg/kg·BW).

**Figure 5:**
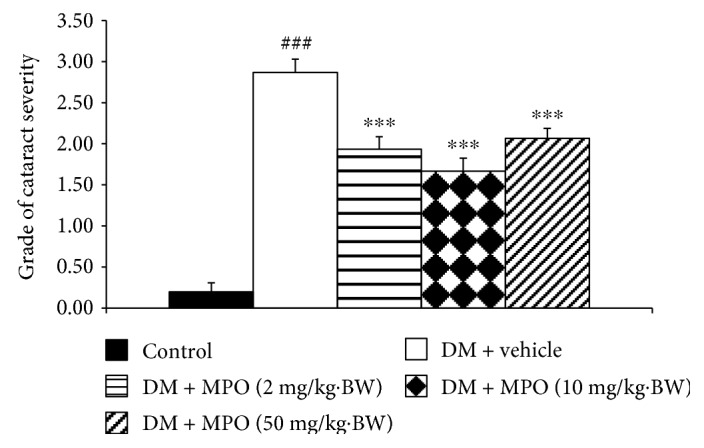
The severity of cataract in control and diabetic rats (DM) which received either vehicle or MPO at doses of 2, 10, and 50 mg/kg·BW at the end of experiment (10 weeks). ^###^*p* value < .001 compared with control and ^∗∗∗^*p* value < .001 compared with diabetic rats which received vehicle (DM + vehicle).

**Figure 6:**
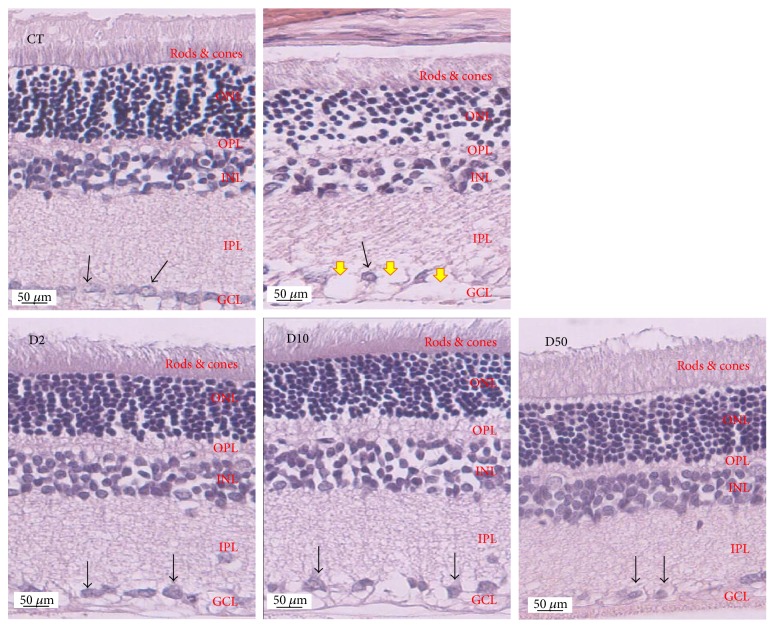
Representative photographs of transverse sections of eye balls (5 *μ*m thickness) showing morphological change of the retina evaluated under light microscope by using H&E staining at the end of a 10-week study period. C: control; V: DM + vehicle; D2: DM + MPO (2 mg/kg·BW); D10: DM + MPO (10 mg/kg·BW); D50: DM + MPO (50 mg/kg·BW); yellow arrow: ganglionic cell loss area; black arrow: ganglion cell.

**Figure 7:**
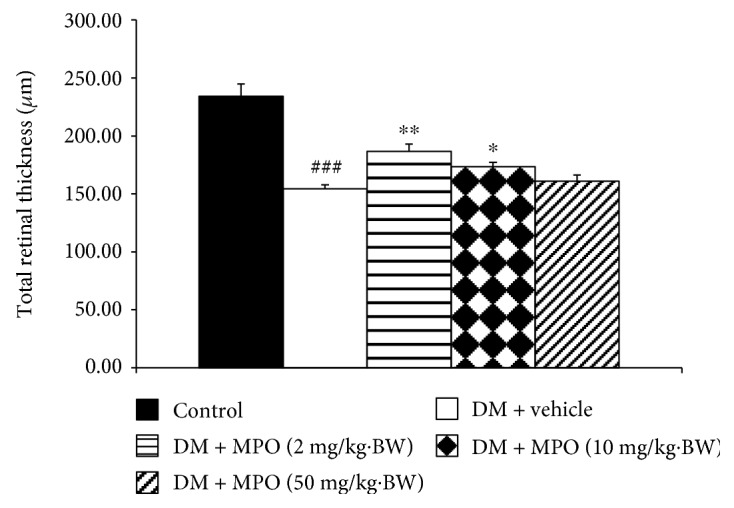
The total retinal thickness (TRT) of control and diabetic rats (DM) which received either vehicle or MPO at doses of 2, 10, and 50 mg/kg·BW at the end of a 10-week experiment period. ^###^*p* value < .001 compared with control and ^∗,∗∗^*p* value < .05 and .01, respectively, compared with diabetic rats which received vehicle (DM + vehicle).

**Figure 8:**
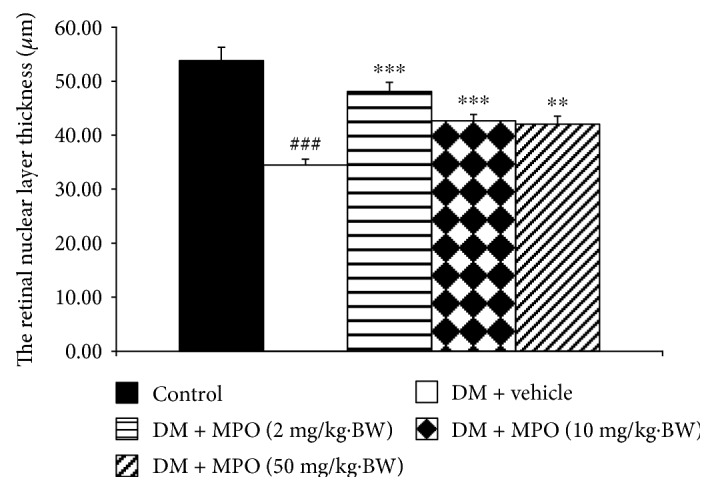
The thickness of the retinal outer nuclear layer (ONL) of control and diabetic rats (DM) which received either vehicle or MPO at doses of 2, 10, and 50 mg/kg·BW at the end of a 10-week experiment period. ^###^*p* value < .001 compared with control and ^∗∗,∗∗∗^*p* value < .01 and .001, respectively, compared with diabetic rats which received vehicle (DM + vehicle).

**Figure 9:**
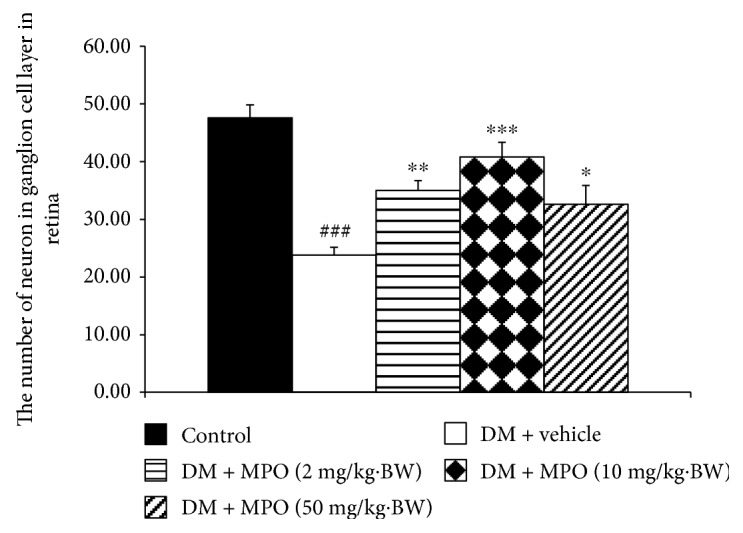
The ganglionic neuron number in the ganglion cell layer (GCL) of the retina of control and diabetic rats (DM) which received either vehicle or MPO at doses of 2, 10, and 50 mg/kg·BW at the end of a 10-week experiment period. ^###^*p* value < .001 compared with control and ^∗,∗∗,∗∗∗^*p* value < .05, 0.01, and .001, respectively, compared with diabetic rats which received vehicle (DM + vehicle).

Figure 4 showed H&E staining of lens sections. It was found that lens epithelial cells, fiber, and cortical architecture of the control group were orderly arranged. Diabetic rats which received vehicle showed the decreased of anterior epithelial cells and the homogenized area was presented. However, the mentioned changes were attenuated in diabetic rats which received MPO.  Figure 5 also showed that the cataract severity in diabetic rats was attenuated in MPO-treated groups compared to that in diabetic rats with vehicle (*p* value < .001 all). Morphological changes of the retina of various groups were also shown in Figure 6. It was found that both inner nuclear cell layer (INL) and outer nuclear cell layer (ONL) of the diabetic rat showed loose cell arrangement. In addition, the ganglion cell in the ganglion cell layer (GCL) of diabetic rats was also considerably decreased when compared to that of the control group. These changes were corresponding with the thickness reduction of the total retina (Figure 7) and outer nuclear cell layer (Figure 8) observed in diabetic rats which received vehicle. Interestingly, Figures 8 and 9 showed that all doses of MPO used in this study attenuated both the decreased thickness in ONL compared to diabetic rats with vehicle (*p* value < .001, .001, and .01, resp.) and the decreased ganglion cell in GCL compared to diabetic rats with vehicle (*p* value < .01, .001, and .05, resp.). However, the attenuation effect on total thickness of retina was observed only in diabetic rats which received MPO at doses of 2 and 10 mg/kg BW compared to diabetic rats with vehicle (*p* value < .01 and .05, resp.) as shown in Figure 7.

### 3.4. Changes of Oxidative Stress Status and Aldose Reductase

Table 2 showed that diabetic rats which received vehicle enhanced both MDA level and AR activity compared to the control group (*p* value < .001 and .01, resp.) but decreased SOD, CAT, and GPx activities compared to the control group (*p* value < .05, .001, and .01, resp.). Diabetic rats which received MPO at a dose of 2 mg/kg BW attenuated the elevation of MDA level and AR activity in rat lens compared to diabetic rats with vehicle treatment (*p* value < .001 and .05, resp.). The elevation of CAT activity in diabetic rats was also mitigated by MPO at this dose compared to diabetic rats with vehicle (*p* value < .01). The increasing dose to 10 mg/kg BW produced the significant reduction of MDA level and AR activity compared to diabetic rats with vehicle (*p* value < .05 all) together with the elevation of CAT and GPx compared to diabetic rats with vehicle (*p* value < .01 and .05, resp.). Diabetic rats which received MPO at a dose of 50 mg/kg significantly decreased AR but increased CAT activities in lens of diabetic rats compared to diabetic rats with vehicle (*p* value < .05 all). No other significant changes were observed.

### 3.5. Effect on MAPK and VEGF

**Figure 10:**
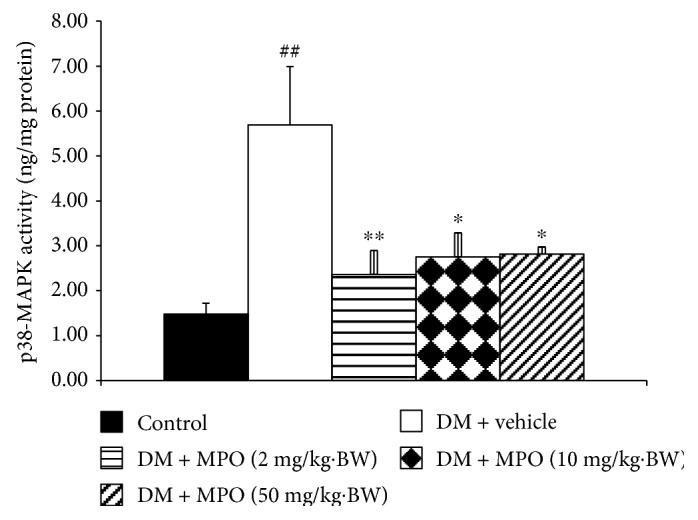
Effect of treatment on p38 MAPK activity in rat lens at the end of a 10-week experimental period. C: control group; DM + vehicle: diabetic rats (DM) which received vehicle; DM + MPO (2 mg/kg·BW): diabetic rats (DM) which received MPO at doses of 2 mg/kg·BW; DM + MPO (10 mg/kg·BW): diabetic rats (DM) which received MPO at doses of 10 mg/kg·BW; DM + MPO (50 mg/kg·BW): diabetic rats (DM) which received MPO at doses of 50 mg/kg·BW (*N* = 10/group). ^##^*p* value < .01 compared with control ^∗,∗∗^*p* value < .05 and .01, respectively, compared with diabetic rats which received vehicle (DM + vehicle).

**Figure 11:**
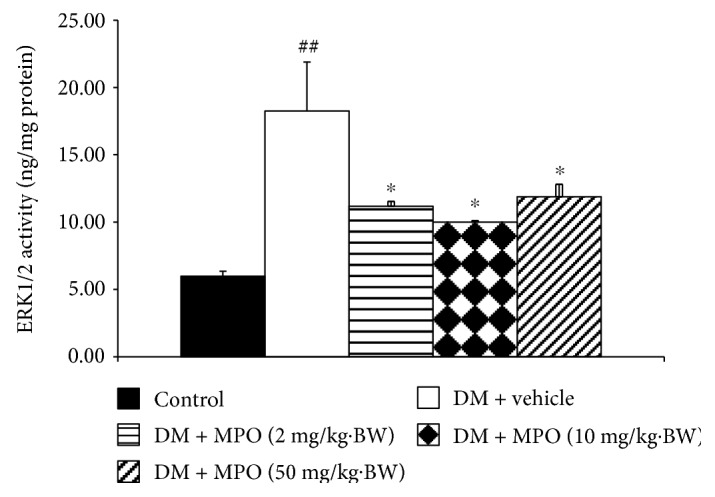
Effect of treatment on ERK1/2 activity in rat lens at the end of a 10-week experimental period. C: control group; DM + vehicle: diabetic rats (DM) which received vehicle; DM + MPO (2 mg/kg·BW): diabetic rats (DM) which received MPO at doses of 2 mg/kg·BW; DM + MPO (10 mg/kg·BW): diabetic rats (DM) which received MPO at doses of 10 mg/kg·BW; DM + MPO (50 mg/kg·BW): diabetic rats (DM) which received MPO at doses of 50 mg/kg·BW (*N* = 10/group). ^##^*p* value < .01 compared with control and ^∗^*p* value < .05 compared with diabetic rats which received vehicle (DM + vehicle).

**Figure 12:**
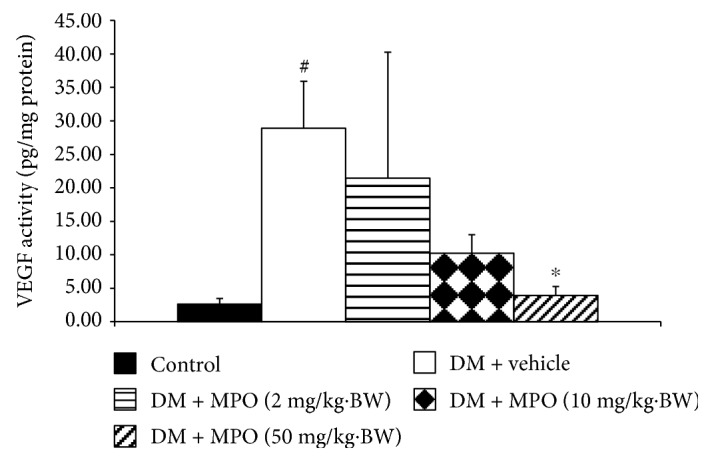
Effect of treatment on VEGF activity in rat lens at the end of a 10-week experimental period. C: control group; DM + vehicle: diabetic rats (DM) which received vehicle; DM + MPO (2 mg/kg·BW): diabetic rats (DM) which received MPO at doses of 2 mg/kg·BW; DM + MPO (10 mg/kg·BW): diabetic rats (DM) which received MPO at doses of 10 mg/kg·BW; DM + MPO (50 mg/kg·BW): diabetic rats (DM) which received MPO at doses of 50 mg/kg·BW (*N* = 10/group). ^#^*p* value < .01 compared with control and ^∗^*p* value < .05 compared with diabetic rats which received vehicle (DM + vehicle).

Diabetic rats increased p38MAPK and ERK1/2 in rat lens as shown in Figures 10 and 11, respectively, compared to the control group (*p* value < .01 and .05). The elevation of p38MAPK activity in STZ-diabetic rat was mitigated by all doses of MPO used in this study compared to diabetic rats with vehicle (*p* value < .01, .05, and .05, resp.). Moreover, the enhanced ERK1/2 in the diabetic rat was also counteracted by MPO at all dosage ranges compared to diabetic rats with vehicle (*p* value < .05 all). VEGF change was also assessed, and results were shown in Figure 12. Diabetic rats showed the increase of VEGF compared to control (*p* value < .05). This change was attenuated only in diabetic rats which received MPO treatment at a dose of 50 mg/kg·BW compared to diabetic rats with vehicle (*p* value < .05).

## 4. Discussion

Oxidative stress has been regarded as an important factor in the pathogenesis of diabetic cataract [20] and retinopathy [6, 21]. The excess oxidative stress presented in diabetic condition can occur by various pathways. High glucose concentration in diabetic condition enhanced the formation of oxygen free radicals such as superoxide anion radicals and hydrogen peroxide. Therefore, the main antioxidant enzymes such as SOD, CAT, and GPx have been used to buffer the oxidative stress more than normal condition resulting in the reduction of all enzymes mentioned earlier in diabetic rats. Unfortunately, the capacity of antioxidant enzymes just mentioned cannot overcome the elevation of oxidative stress. Therefore, an excess oxidative stress attacks the cell components especially the polyunsaturated fatty acids (PUFA) leading to the increase level of MDA, a lipid peroxidation product [22]. The impairment in the oxidant/antioxidant equilibrium also induces tissue damage and diabetic complications [23]. Hyperglycemia induces auto-oxidation of glucose [24] and enhances the formation of advanced glycation end products (AGE) via the nonenzymatic reaction [25]. Then, these processes in turn give rise to the excess oxidative stress. It has been reported that the increased oxidative stress induced by AGE can occur via either the increased production [26] or the decreased antioxidant capability [27]. In addition, hyperglycemia has been reported to induce electron transport chain dysfunction, resulting in electron leakage through complex I and complex III of mitochondria leading to the increased oxidative stress [28]. Moreover, hyperglycemia also increases aldose reductase-dependent polyol pathway giving rise to the increased oxidative stress [29]. The elevation of both aldose reductase and oxidative stress mentioned above was also in agreement with the findings in our study. It has been reported that aldose reductase also plays a major role in diabetes-induced oxidative stress in the lens [30] and in the pathophysiology of diabetic retinopathy [31]. The suppression of aldose reductase can attenuate not only an excess oxidative stress status in the lens but also osmotic swelling, ionic imbalance, and protein insolubilization induced by sorbitol, a product in the polyol pathway [32, 33]. In addition, aldose reductase inhibitor can also improve diabetic retinopathy [34]. Therefore, it has been considered as the potential therapeutic target for both diabetic cataract and diabetic retinopathy [22, 35]. Recent data also demonstrate that both polyol accumulation [36] and oxidative stress [36–38] activate the expression of mitogen-activated protein kinases (MAPKs), a signal transduction pathway contributing an important role in the survival of lens epithelium [36] and diabetic retinopathy [39]. In addition to both mentioned parameters, MAPKs can also be stimulated by growth factors such as vascular endothelial growth factor (VEGF) [36].

In this study, diabetic rats which received vehicle also showed the elevation of various parameters mentioned earlier including MDA level, aldose reductase, MAPK expression especially p38 mitogen-activated protein kinases (p38 MAPK), and extracellular signal-related protein kinase (ERK) together with the increased VEGF in rat lens. However, SOD, CAT, and GPx activities were decreased in diabetic rats which received vehicle. These changes were in agreement with the previous studies [22, 23].

Interestingly, diabetic rats which received MPO at all doses used in this study showed the reduction of aldose reductase activity and the expression of both p38MAPK and ERK1/2 in rat lens together with the improved both diabetic cataract and diabetic retinopathy. Both low and medium doses of MPO could decrease MDA level while only the medium dose of MPO produced a significant increased GPx activity in the lens of diabetic rats. Taken all data together, the anticataractogenesis in diabetic rats induced by MPO might occur partly via the suppression of aldose reductase giving rise to the decreased protein insolubilization induced by sorbitol resulting in improved lens opacity. In addition, the decreased oxidative stress either by the increased CAT or by the increased GPx activities also contributed the role. MPO could also exert the effect via the decreased expression of both p38MAPK and ERK induced by aldose reductase and oxidative stress leading to the reduction of lens epithelium apoptosis. Since all parameters mentioned earlier together with the increased VEGF expression in rat lens also play the essential roles in the pathophysiology of diabetic retinopathy, the suppression of the parameters just mentioned might contribute to the roles in the antiretinopathy effect of MPO in diabetic rats. Both oxidative stress and the aldose reductase might exert antiretinopathy partly via the mechanism as same as that of anticataractogenesis. The decreased p38MAPK and ERK which in turn decrease the apoptosis of various cell types especially ganglion cells in the retina leading to the improved retinopathy might also play a role. Moreover, MPO at high dose might also suppress VEGF activity leading to the decreased metalloproteinase (MMP) stimulation and decreased retina damage [40].

However, our data failed to show the tight relationship either between the improved diabetic cataract and oxidative stress status or between the improved diabetic retinopathy and oxidative stress status in diabetic rats treated with MPO. The improved lens opacity and diabetic retinopathy in diabetic rats were attenuated by the combination extract of mango seed and the aerial parts of Vietnamese coriander (MPO) at all dosages used in this study whereas the decreased MDA level was observed only in diabetic rats which received MPO at doses of 2 and 10 mg/kg·BW. The lack of close relationship between aldose reductase and MDA may occur because oxidative stress can be generated by various pathways mentioned earlier. Although the decreased blood sugar could also improve diabetic complications, our data revealed that the improved diabetic cataract and diabetic retinopathy induced by MPO might not relate to the decreased blood sugar level. During this decade, several studies have demonstrated that polyphenol-rich substances can improve diabetic cataract and diabetic retinopathy [10, 40, 41]. It has been demonstrated that the protective effects against both conditions of polyphenol-rich substances are attribute to antioxidant and aldose reductase inhibitory effects of their phenolic compounds [8]. Therefore, the anticataractogenesis and antiretinopathy of MPO observed in this study might be associated with phenolic compounds presented in MPO or may be associated with the interaction effects of various ingredients presented in MPO. Further exploration is required to provide the precise understanding concerning this issue. Interestingly, no side effects were observed throughout the study period although MPO exerts its influence at many targets simultaneously.

## 5. Conclusions

This study has clearly shown that the combination extract of mango seed and aerial parts of Vietnamese coriander or MPO is the potential candidate to prevent eye complications such as cataract and retinopathy in diabetes mellitus. The possible underlying mechanism might occur via the suppression of aldose reductase, oxidative stress status, and MAPK signal transduction especially p38MAPK and ERK together with the suppression of VEGF. Since diabetic complications are complex, the multitarget protective agent has gained much attention due to its simultaneous action at multitargets. However, the determination of possible active ingredient(s) is still required further exploration.

## Figures and Tables

**Table 1:** Opacity index of the lens of control and diabetic rats (DM) which received either vehicle or MPO at doses of 2, 10, and 50 mg/kg·BW for 10 weeks (*N* = 10/group). ^#,##^*p* value < .05 and .01, respectively, compared with control group and ^∗,∗∗,∗∗∗^*p* value < .05, .01, and .001, respectively, compared with diabetic rats which received vehicle (DM + vehicle).

Time duration	Opacity index of the lens (mean ± SEM)				
	Control	DM + vehicle	DM + MPO (2 mg/kg·BW)	DM + MPO (10 mg/kg·BW)	DM + MPO (50 mg/kg·BW)
Baseline	0.00 ± 0.00	0.00 ± 0.00	0.00 ± 0.00	0.00 ± 0.00	0.00 ± 0.00
Week 1	0.00 ± 0.00	0.00 ± 0.00	0.00 ± 0.00	0.00 ± 0.00	0.00 ± 0.00
Week 2	0.00 ± 0.00	0.25 ± 0.08^#^	0.10 ± 0.06	0.13 ± 0.06	0.21 ± 0.08
Week 3	0.00 ± 0.00	0.42 ± 0.12^#^	0.50 ± 0.09	0.53 ± 0.09	0.54 ± 0.10
Week 4	0.00 ± 0.00	1.00 ± 0.12^##^	0.67 ± 0.09^∗^	0.87 ± 0.06	0.71 ± 0.09^∗^
Week 5	0.00 ± 0.00	1.46 ± 0.12^##^	0.87 ± 0.06^∗∗∗^	1.00 ± 0.00^∗∗∗^	0.86 ± 0.07^∗∗∗^
Week 6	0.00 ± 0.00	2.04 ± 0.18^##^	1.23 ± 0.14^∗∗∗^	1.37 ± 0.11^∗∗∗^	1.39 ± 0.16^∗∗∗^
Week 7	0.08 ± 0.08	2.50 ± 0.17^##^	1.70 ± 0.17^∗∗∗^	1.77 ± 0.13^∗∗∗^	1.71 ± 0.21^∗∗∗^
Week 8	0.17 ± 0.11	3.17 ± 0.20^##^	2.37 ± 0.14^∗∗∗^	2.17 ± 0.17^∗∗∗^	2.57 ± 0.23^∗∗∗^
Week 9	0.42 ± 0.15	3.67 ± 0.10^##^	3.03 ± 0.13^∗∗^	2.81 ± 0.21^∗∗∗^	3.04 ± 0.19^∗∗^
Week 10	0.42 ± 0.15	4.00 ± 0.00^##^	3.64 ± 0.11^∗^	3.38 ± 0.16^∗∗∗^	3.61 ± 0.11^∗^

**Table 2:** Comparison of aldose reductase, malondialdehyde (MDA), catalase (CAT), glutathione peroxidase (GPx), and superoxide dismutase (SOD) in lens of all groups. C: control group; DM + vehicle: diabetic rats (DM) which received vehicle; DM + MPO (2 mg/kg·BW): diabetic rats (DM) which received MPO at doses of 2 mg/kg·BW; DM + MPO (10 mg/kg·BW): diabetic rats (DM) which received MPO at doses of 10 mg/kg·BW; DM + MPO (50 mg/kg·BW): diabetic rats (DM) which received MPO at doses of 50 mg/kg·BW (*N* = 10/group). ^#,##,###^*p* value < .05, .01, and .001, respectively, compared with control group and ^∗,∗∗,∗∗∗^*p* value < .05, .01, and .001, respectively, compared with diabetic rats which received vehicle (DM + vehicle).

Group	MDA (U/mg protein)	CAT (U/mg protein)	GPx (U/mg protein)	SOD (U/mg protein)	AR (nmol/min/mg)
Control	0.52 ± 0.08	19.55 ± 0.59	1.01 ± 0.11	8.98 ± 0.34	0.005 ± 0.001
DM + vehicle	1.06 ± 0.10^###^	13.04 ± 0.84^###^	0.62 ± 0.04^##^	6.83 ± 0.87^#^	0.013 ± 0.004^##^
DM + MPO (2 mg/kg·BW)	0.73 ± 0.03^∗∗∗^	17.91 ± 0.55^∗∗^	0.80 ± 0.10	8.06 ± 0.36	0.007 ± 0.001^∗^
DM + MPO (10 mg/kg·BW)	0.87 ± 0.05^∗^	17.69 ± 0.92^∗∗^	0.85 ± 0.01^∗^	7.28 ± 0.43	0.008 ± 0.001^∗^
DM + MPO (50 mg/kg·BW)	0.98 ± 0.05	16.61 ± 1.35^∗^	0.71 ± 0.04	6.10 ± 0.52	0.008 ± 0.001^∗^

## References

[1] Gürler B., Vural H., Yilmaz N., Oguz H., Satici A., Aksoy N. (2000). The role of oxidative stress in diabetic retinopathy. *Eye (London, England)*.

[2] Kowluru R. A. (2003). Effect of reinstitution of good glycemic control on retinal oxidative stress and nitrative stress in diabetic rats. *Diabetes*.

[3] Madsen-Bouterse S. A., Kowluru R. A. (2008). Oxidative stress and diabetic retinopathy: pathophysiological mechanisms and treatment perspectives. *Reviews in Endocrine & Metabolic Disorders*.

[4] Kowluru R. A., Abbas S. N. (2003). Diabetes-induced mitochondrial dysfunction in the retina. *Investigative Ophthalmology & Visual Science*.

[5] Klettner A., Roider J. (2009). Constitutive and oxidative-stress-induced expression of VEGF in the RPE are differently regulated by different mitogen-activated protein kinases. *Graefe's Archive for Clinical and Experimental Ophthalmology*.

[6] Kowluru R. A., Chan P. S. (2007). Oxidative stress and diabetic retinopathy. *Experimental Diabetes Research*.

[7] Obrosova I. G., Pacher P., Szabó C. (2005). Aldose reductase inhibition counteracts oxidative-nitrosative stress and poly (ADP-ribose) polymerase activation in tissue sites for diabetic complications. *Diabetes*.

[8] Pandita N. S., Vaidya A. S. (2014). Therapeutic potential of plant phenolics for the management of diabetic retinopathy. *Pharmaceutical Crops*.

[9] Thiraphatthanavong P., Wattanathorn J., Muchimapura S. (2014). Preventive effect of *Zea mays* L. (purple waxy corn) on experimental diabetic cataract. *BioMed Research International*.

[10] Stefek M. (2011). Natural flavonoids as potential multifunctional agents in prevention of diabetic cataract. *Interdisciplinary Toxicology*.

[11] Arikan S., Ersan I., Karaca T. (2015). Quercetin protects the retina by reducing apoptosis due to ischemia-reperfusion injury in a rat model. *Arquivos Brasileiros de Oftalmologia*.

[12] Suryanarayana P., Saraswat M., Mrudula T., Krishna T. P., Krishnaswamy K., Reddy G. B. (2005). Curcumin and turmeric delay streptozotocin-induced diabetic cataract in rats. *Investigative Opthalmology & Visual Science*.

[13] Ohkawa H., Ohishi N., Yagi K. (1979). Assay for lipid peroxides in animal tissues by thiobarbituric acid reaction. *Analytical Biochemistry*.

[14] Sun Y., Oberley L. W., Li Y. (1988). A simple method for clinical assay of superoxide dismutase. *Clinical Chemistry*.

[15] Goth L. (1991). A simple method for determination of serum catalase activity and revision of reference range. *Clinica Chimica Acta*.

[16] Rotruck J. T., Pope A. L., Ganther H. E., Swanson A. B., Hafeman D. G., Hoekstra W. G. (1973). Selenium: biochemical role as a component of glutathione peroxidase. *Science*.

[17] Patel M., Mishra S. (2009). Aldose reductase inhibitory activity and anticataract potential of some traditionally acclaimed antidiabetic medicinal plants. *Oriental Pharmacy and Experimental Medicine*.

[18] Agarwal R., Iezhitsa I., Awaludin N. A. (2013). Effects of magnesium taurate on the onset and progression of galactose-induced experimental cataract: in vivo and in vitro evaluation. *Experimental Eye Research*.

[19] Martin P. M., Roon P., Ells T. K. V., Ganapathy V., Smith S. B. (2004). Death of retinal neurons in streptozotocin-induced diabetic mice. *Investigative Ophthalmology & Visual Science*.

[20] Pollreisz A., Schmidt-Erfurth U. (2010). Diabetic cataract-pathogenesis, epidemiology and treatment. *Journal of Ophthalmology*.

[21] Anderson R. E., Rapp L. M., Wiegand R. D. (1984). Lipid peroxidation and retinal degeneration. *Current Eye Research*.

[22] Ozmen B., Ozmen D., Erkin E., Guner I., Habif S., Bayindir O. (2002). Lens superoxide dismutase and catalase activities in diabetic cataract. *Clinical Biochemistry*.

[23] Halliwell B. (1994). Free radicals, antioxidants, and human disease: curiosity, cause, or consequence?. *Lancet*.

[24] Wolff S. P., Dean R. T. (1987). Glucose autoxidation and protein modification. The potential role of “autoxidative glycosylation” in diabetes. *Biochemical Journal*.

[25] Mullarkey C. J., Edelstein D., Brownlee M. (1990). Free radical generation by early glycation products: a mechanism for accelerated atherogenesis in diabetes. *Biochemical and Biophysical Research Communications*.

[26] Schmidt A. M., Hori O., Brett J., Yan S. D., Wautier J. L., Stern D. (1994). Cellular receptors for advanced glycation end products. Implications for induction of oxidant stress and cellular dysfunction in the pathogenesis of vascular lesions. *Arteriosclerosis, Thrombosis, and Vascular Biology*.

[27] Morgan P. E., Dean R. T., Davies M. J. (2002). Inactivation of cellular enzymes by carbonyls and protein-bound glycation/glycoxidation products. *Archives of Biochemistry and Biophysics*.

[28] Murphy M. P. (2009). How mitochondria produce reactive oxygen species. *Biochemical Journal*.

[29] Drel V. R., Pacher P., Ali T. K. (2008). Aldose reductase inhibitor fidarestat counteracts diabetes-associated cataract formation, retinal oxidative-nitrosative stress, glial activation, and apoptosis. *International Journal of Molecular Medicine*.

[30] Chung S. S., Ho E. C., Lam K. S., Chung S. K. (2003). Contribution of polyol pathway to diabetes-induced oxidative stress. *Journal of the American Society of Nephrology*.

[31] Obrosova I. G., Kador P. F. (2011). Aldose reductase/polyol inhibitors for diabetic retinopathy. *Current Pharmaceutical Biotechnology*.

[32] Kinoshita J. H., Fukushi S., Kador P., Merola L. O. (1979). Aldose reductase in diabetic complications of the eye. *Metabolism*.

[33] Kinoshita J. H., Kador P., Catiles M. (1981). Aldose reductase in diabetic cataracts. *Journal of the American Medical Association*.

[34] Sun W., Oates P. J., Coutcher J. B., Gerhardinger C., Lorenzi M. (2006). A selective aldose reductase inhibitor of a new structural class prevents or reverses early retinal abnormalities in experimental diabetic retinopathy. *Diabetes*.

[35] Roy S., Kern T. S., Song B., Stuebe C. (2017). Mechanistic insights into pathological changes in the diabetic retina: implications for targeting diabetic retinopathy. *American Journal of Pathology*.

[36] Zatechka D. S., Kador P. F., Garcia-Castineiras S., Lou M. F. (2003). Diabetes can alter the signal transduction pathways in the lens of rats. *Diabetes*.

[37] Krysan K., Lou M. F. (2002). Regulation of human thioltransferase (hTTase) gene by AP-1 transcription factor under oxidative stress. *Investigative Ophthalmology & Visual Science*.

[38] Purves T., Middlemas A., Agthong S. (2001). A role for mitogen-activated protein kinases in the etiology of diabetic neuropathy. *FASEB Journal*.

[39] Du Y., Tang J., Li G. (2010). Effects of p38 MAPK inhibition on early stages of diabetic retinopathy and sensory nerve function. *Investigative Ophthalmology & Visual Science*.

[40] Gupta N., Mansoor S., Sapkal A. (2013). Diabetic retinopathy and VEGF. *Ophthalmology Journal*.

[41] Ghosh D., Konishi T. (2007). Anthocyanins and anthocyanin-rich extracts: role in diabetes and eye function. *Asia Pacific Journal of Clinical Nutrition*.

